# Diagnostic role of inflammatory markers in pediatric Brucella arthritis

**DOI:** 10.1186/s13052-016-0211-5

**Published:** 2016-01-11

**Authors:** Fesih Aktar, Recep Tekin, Mehmet Selçuk Bektaş, Ali Güneş, Muhammet Köşker, Sabahattin Ertuğrul, Kamil Yılmaz, Kamuran Karaman, Hasan Balık, İlyas Yolbaş

**Affiliations:** Department of Pediatric Infectious Disease, Dicle University School of Medicine, 21280 Diyarbakir, Turkey; Department of Clinical Microbiology and Infectious Disease, Dicle University School of Medicine, Diyarbakir, Turkey; Department of Pediatric, Van Research Hospital, Van, Turkey; Department of Pediatric Infectious Disease, Diyarbakir Children’s Hospital, Diyarbakir, Turkey; Department of Pediatric, Dicle University School of Medicine, Diyarbakir, Turkey; Department of Pediatric, Diyarbakir Children’s Hospital, Diyarbakir, Turkey; Department of Pediatric, Yüzüncü Yil University School of Medicine, Van, Turkey

**Keywords:** Brucella arthritis, Child, Correlation, Inflammatory markers, Diagnostic role

## Abstract

**Background:**

As a multisystem infectious disease, there is an inflammation, which causes increase in acute phase reactants in brucellosis. The mean platelet volume (MPV), platelet distribution width (PDW), red cell distribution width (RDW), neutrophil to lymphocyte ratio (NLR) and platelet to lymphocyte ratio (PLR) have been identified as markers of inflammation. The present study aimed to evaluate diagnostic values of these biomarkers in brucella arthritis (BA).

**Methods:**

The study included 64 children with BA and 66 healthy control subjects. Demographic features, joint involvement, erythrocyte sedimentation rate (ESR), C-reactive protein (CRP) and hematological variables were retrospectively recorded. In addition, results of synovial fluid and serum tube agglutination test for brucella together with treatment regimens were recorded.

**Results:**

The mean age of the patients (53.1 % male) was 92.3 ± 41.2 months. The most commonly affected joint was ankle (53.1 %). Synovial fluid puncture-brucella agglutination test was positive in 22 (34.3 %) patients. Puncture culture was positive in 9 patients. Most of the patients (57.8 %) were treated with a combination of rifampicin plus sulfamethoxazole/trimethoprim and gentamicin. Significantly higher mean PDW, RDW, MPV, NLR and PLR values were found in children with BA compared to control subjects (*p* < 0.05). A positive correlation was found between MPV and NLR values (*R*^2^ = 0.192, *p* < 0.001).

**Conclusion:**

Our findings indicated that NLR and PLR are indirect markers of inflammation that may be observed abnormally increased in children with brucella arthritis. Further longitudinal studies are needed to investigate this topic to establish the more clear associations.

## Background

Brucellosis, the most common bacterial zoonosis in the world, is still endemic in many developing countries. The clinical presentation of brucellosis is non-specific and the course and the severity of infection is variable; in humans, it presents as a multisystem disease involving many organs and tissues [1]. Fever and arthritis are the most common signs. Osteoarticular involvement is one of the most frequent complications of brucellosis. Although in adults with osteoarticular brucellosis due to Brucella abortus from Northwestern Spain sacroiliitis and spondylitis were more common than peripheral arthritis [2], monoarthritis is now considered as the predominant musculoskeletal manifestation of brucellosis [3, 4]. The most commonly affected joints are the hip and the knee. Unlike in adults, the sacroiliac joint and the axial skeleton are rarely affected. Monoarthritis is more common than polyarthritis. This may lead to confusion with pyogenic arthritis in children; therefore, in a community where *brucella* is common, awareness about this entity should prompt the investigation of this disease, and physicians should have a high index of suspicion for brucella arthritis (BA) [5].

Laboratory findings may be normal in some pediatric cases of brucella arthritis; however, it is not possible to take synovial fluid from all of these patients. Therefore, new inflammatory markers are required in diagnosis of pediatric BA patients. There are few previous studies on the parameters indicating new inflammatory markers in pediatric brucella arthritis. The present study aimed to investigate the levels of MPV, PDW, RDW, NLR and PLR as possible indirect inflammatory markers in children with brucella arthritis.

## Methods

This retrospective study was performed by the two center of medical faculty of university pediatric clinics. The medical records of all patients with BA between November 2011 and January 2014 were obtained from the hospital records. A total of 64 children with BA and 66 age- and gender-matched healthy controls were enrolled in the study.

Healthy subjects were children who applied to hospital for routine check-up or for preoperative evaluation of minor elective surgery such as circumcision or hernia repair. Control group subjects were recruited from hospital records of these children. Children with any sign of infection or systemic illness were excluded from the control group.

Arthritis occurred for the first time in all patients within the week before admission to hospital. The diagnosis of arthritis was made if the subjects had joint pain, restriction of movement, and swelling. Swelling was not essential for the diagnosis of hip, spine, or sacroiliac arthritis. Although encountered in many cases, additional signs such as effusion, redness and increased temperature on joint were not considered essential for the diagnosis of arthritis.

The diagnosis of brucellosis with joint involvement was established according to the presence of all of the following criteria; a clinical picture compatible with arthritis, isolation of *Brucella* from blood or synovial fluids, positive *brucella* serology test 1:≥160, using the Standard Agglutination Test (SAT) for patients presenting with symptoms suggestive of brucellosis. For screening and in the absence of clinical indicators of active brucellosis, a titer of 1:320 or higher is more specific for the presence of the disease. Pediatric patients with a synovial fluid culture positive for brucella and available results of the cytological examination of the synovial fluid aspirate were identified. Relevant demographic, clinical and laboratory data, and treatment modalities and outcomes were obtained from patients’ follow-up cards and hospital records.

Hemogram parameters including white blood cell (WBC) count, neutrophil count, lymphocyte count, hemoglobin (Hb), platelet count (PLT), platelet distribution width (PDW), red cell distribution width (RDW), mean platelet volume (MPV), neutrophil to lymphocyte ratio (NLR) and platelet to lymphocyte ratio (PLR) were assessed. C-reactive protein (CRP), erythrocyte sedimentation rate (ESR), agglutination assay and culture for both blood and joint puncture at admission were also recorded.

NLR and PLR were calculated as the ratio of neutrophils to lymphocytes and platelets to lymphocytes, respectively. These hematological variables were measured and recorded in the healthy control subjects as well. Comparison between the study and the control subjects was performed with regards to WBC, neutrophil count, lymphocyte count, PDW, RDW, PLT, MPV, NLR and PLR. Blood samples were obtained using a vacutainer and collected in tubes containing standard EDTA. All blood samples were tested for hematological parameters using the same regularly calibrated analyzer (Abbott CELL-DYN 3700, United States).

Joint fluid was aspirated from the affected joint following a strict sterile technique. Since usually only small amounts of fluid were obtained, the synovial fluid specimens were only sent for cytological and bacteriological examination, and tested for antibrucella antibodies using microagglutination test.

WBC, Hb, neutrophil count, lymphocyte count, PLT, MPV, NLR and PLR values were compared between the study and the control groups.

Patients with a clear-cut underlying pathology like various bone and joint diseases, connective tissue, rheumatic disorders, chronic disorders, anemia or other hematological diseases, acute bacterial infection as well as fever of other etiologies, who were over 18 years old and whose file records were inaccessible, were excluded from the study.

The Non-Interventional Clinical Ethics Committee of Dicle University Medical Faculty approved the study protocol.

### Statistical analysis

The normality of data distribution was determined using the Kolmogorov-Smirnov test. Normally distributed numerical variables were expressed in mean plus/minus standard deviation. Normally distributed numeric variables were compared using the Student’s *t*-test or One-way ANOVA test. Data corresponding to an abnormal distribution were compared using the non-parametric Mann–Whitney *U*-test or Kruskal-Wallis test. The Chi-square test was used to compare categorical variables between the groups. Correlations between numerical variables were evaluated using Pearson’s or Spearman’s correlation analysis. P-values of less than 0.05 were considered statistically significant. The data were analyzed using Statistical Package for Social Sciences (SPSS) version 18.0 program for Windows.

## Results

The mean age of the patients was 92.3 ± 41.2 months and 53.1 % (*n* = 34) of the patients were male. The mean age of the control group was 98.5 ± 44.0 months and 53 % (*n* = 35) were male. There were no significant differences in the mean age and gender distribution between the study and the control groups (*p* > 0.05).

The median duration of hospitalization was 6 (2–76) days in patients with BA. Five (7.8 %) patients had hepatomegaly, 15 (23.4 %) had splenomegaly and five (7.8 %) had hepatosplenomegaly. The most common symptoms at admission were arthralgia, joint pain and fever. The most commonly affected joint was ankle (*n* = 34, 53.1 %). Most of the patients (*n* = 37, 57.8 %) were treated with a combination of rifampicin plus sulfamethoxazole/trimethoprim and gentamicin. Synovial fluid puncture was performed in 24 (37.5 %) patients and brucella agglutination test was found positive in 22 (34.3 %) patients. Puncture culture was positive in nine (14 %) patients. The average ESR was 40.9 ± 20.4 mm/h and the mean CRP level was 28.1 ± 22.4 mg/L in patients with BA (Table 1).

**Table 1:** Demographic and clinical characteristics and treatment modalities in patients with Brucella arhtritis

Clinical features	Mean ± SD, median range or number (%) (n:64)
Mean age (month)	92.3 ± 41.2
Gender	
Male	34 (53.1)
Female	30 (46.9)
Duration of hospitalization (day)	6 (2–76)
Symptoms and clinical findings	
Arthralgia	64 (100)
Joint pain	61 (95.3)
Fever	55 (85.9)
Myalgia	49 (76.6)
Anorexia	46 (71.9)
Weakness	40 (62.5)
Shivering	39 (60.9)
Restriction of movement	36 (56.2)
Diaphoresis	33 (51.6)
Swelling	22 (34.3)
Increase temperature on joint	22 (34.3)
Joint redness	22 (34.3)
Headache	20 (31.3)
Abdominal pain	14 (21.9)
Joint effusion	13 (20.3)
Chest pain	11 (17.2)
Neck pain	2 (3.1)
Joint involvement	
Ankle	34 (53.1)
Knee	30 (46.9)
Laboratory findings	
Erythrocyte sedimentation rate (mm/h)	40.9 ± 20.4
C-reactive protein (mg/L)	28.1 ± 22.4
Brucella hemagglutination test	
160	10 (15.8)
320	25 (39.1)
≥640	29 (45.3)
Positive culture	
Blood	4 (6.3)
Joint puncture	4 (6.3)
Blood + Joint puncture	5 (7.9)
Treatment	
Rifampicin + Tetracycline + Streptomycin	13 (20.3)
Rifampicin + Tetracycline + Gentamicin	14 (21.8)
Rifampicin + TMP-SMX + Gentamicin	37 (57.8)

*TMP-SMX* trimethoprim-sulfamethoxazole, *SD* standard deviation

**Fig. 1:**
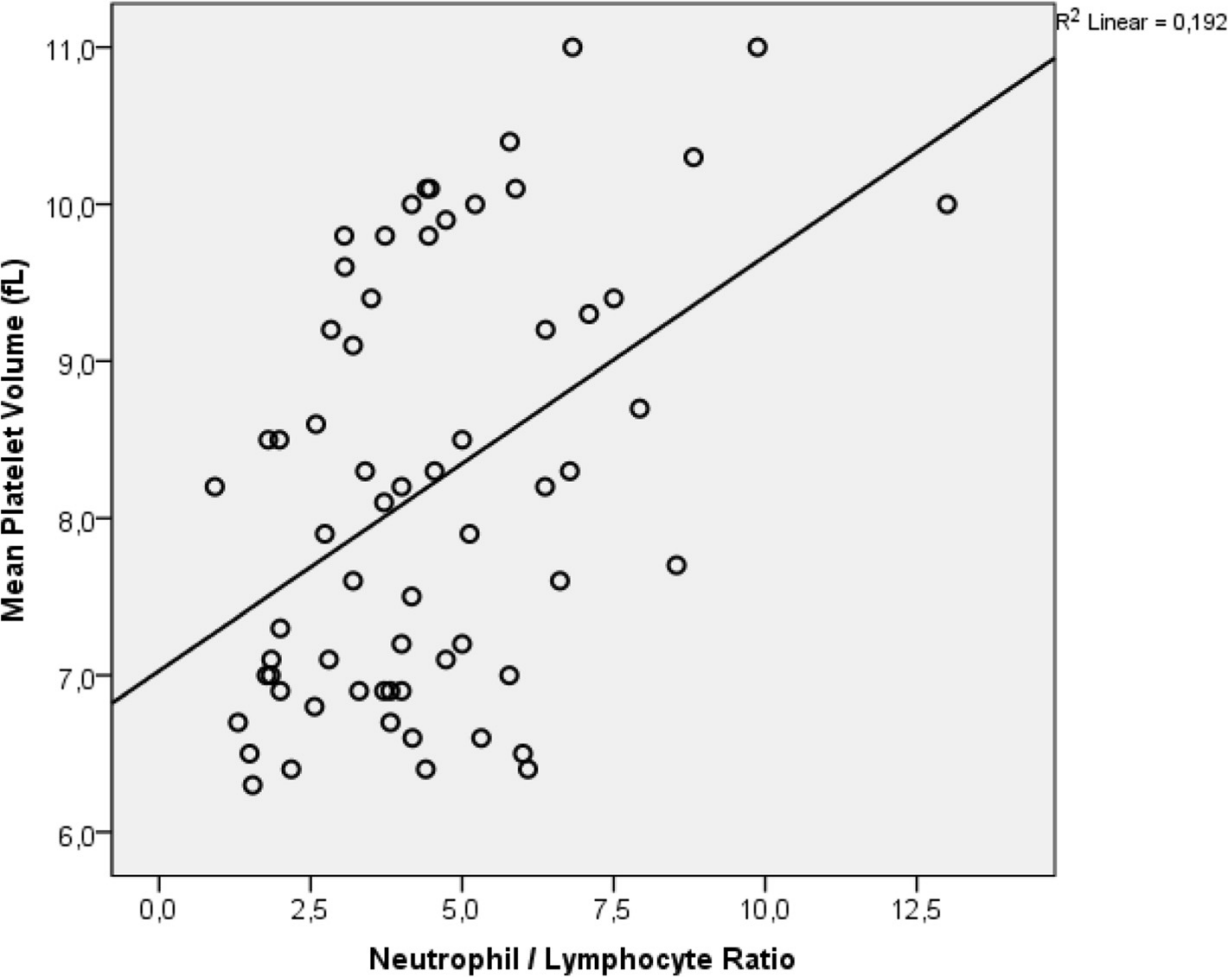
The relationship between mean platelet volume and neutrophil to lymphocyte ratio

Median WBC was 10.0 ± 4.2 × 10^3^/ml, PDW was ± 3.0, RDW was 16.2 ± 1.4, MPV value was 8.2 fL (6.3–11), NLR was 4.1 (1–13) and PLR was 154 (9.3–497) in the patient group. The patient group had significantly higher WBC, neutrophil count, PDW, RDW, MPV, NLR, PLR and lower lymphocyte and platelet counts at admission, compared to the control group (*p* < 0.05) (Table 2). There was a positive correlation between MPV and NLR (*R*^2^ = 0.192, *p* < 0.001) (Fig. 1). No correlation was found between MPV, ESR and CRP values.

**Table 2:** Laboratory findings of the study and control groups

Parameters	Study group mean ± SD or median range	Control group mean ± SD or median range	*p value*
WBC (K/μL)	10.0 ± 4.2	8.25 ± 1.7	0.040
Neutrophil (10^3^/μL)	7.10 (1.70-16.5)	3.98 (2.20-9.12)	<0.001
Lymphocyte (10^3^/μL)	1.60 (0.60-7.0)	2.92 (0.73-4.95)	<0.001
Platelet count (K/μL)	282 ± 144	314 ± 65.7	<0.001
PDW,%	38,1 ± 3.0	23.0 ± 3.9	<0.001
RDW,%	16.2 ± 1.4	9.4 ± 1.8	<0.001
MPV (fl)	8.2 (6.3-11)	7.3 (5.7-12.3)	0.026
NLR	4.1 (1–13)	1.4 (0.6-10.8)	<0.001
PLR	154 (9.3-497)	106 (50–525)	<0.001

*WBC* white blood cell, *PDW* platelet distribution width, *RDW* red cell distribution width, *MPV* mean platelet volume, *NLR* neutrophil to lymphocyte ratio, *PLR* platelet to lymphocyte ratio, *SD* standart deviation

## Discussion

Although hematological changes are common in brucella arthritis, they are not diagnostic and usually do not require treatment. In childhood brucella arthritis, hematological disorders may occur as leukocytosis, anemia, relative lymphocytosis along with leukopenia, thrombocytopenia and pancytopenia [6]. The study by El-Koumi et al. found anemia in 43 %, leukopenia in 38 %, leukocytosis in 20 % and pancytopenia in 18 % of the cases [7]. Similar to the previous studies, the present study found anemia in 45.3 %, thrombocytopenia in 21.8 %, leukopenia in 10.9 %, leukocytosis in 9.3 % and pancytopenia in 7.8 % of the patients. Significantly higher leukocyte and neutrophil counts were found in brucellosis patients compared to the control group, whereas the lymphocyte and thrombocyte counts were lower.

The present study aimed to investigate the predictive contribution value of NLR, PLR and MPV in the diagnosis of BA. Our findings showed that NLR, PLR and MPV were higher in patients with BA compared to the control group.

NLR can be determined from routine blood differentials at no additional cost. Changes in the relative abundance of leukocyte subgroups occur in parallel with the increase in overall leukocyte count. Lymphocyte count decreases when neutrophil count increases. NLR increases in inflammatory conditions and this increase is considered as an indicator of systemic inflammation [8]. Studies showed that platelets also play an active role in inflammation, while having regulatory effects on the immune system [9]. The study by Günes et al. [10] was conducted on patients with juvenil rheumatoid arthritis (JRA) and demonstrated that NLR was higher in patients with juvenile idiopathic arthritis compared with the control group. In another study, significantly higher NLR values were found in patients with ankylosing spondylitis [11]. In the study by Türkmen et al., the PLR ratio showed better performance than the NLR ratio in the prediction of inflammation in patients with end-stage renal disease [12]. As a result of changes caused by the inflammation in neutrophils, platelets and lymphocytes, NLR and PLR have turned into inflammatory markers. Based on the results of the present study and other similar studies, we suggest that the NLR and PLR ratios may be inflammatory markers that can be used in the diagnosis and follow-up of the disease in children with brucella arthritis.

Hematologic abnormalities are observed in brucellosis. One of these abnormalities is thrombocytopenia. Over release of proinflammatory cytokines and acute-phase reactants can suppress the size of platelets [8, 9]. The study by Okan et al. found that MPV was statistically significantly lower in brucellosis cases compared to control group [9]. Küçükbayrak et al. and Bozkurt et al. conducted studies on adult patients and established that MPV was increased statistically after treatment in brucellosis cases [13, 14]. The literature contains many studies regarding several diseases related to MPV, PDW and RDW, and a part of such studies demonstrated increased MPV in acute coronary syndrome, diabetes mellitus, cerebrovascular conditions, preeclampsia, renal artery stenosis, hypercholesterolemia, smoking and sepsis [15–17]. However, there are few studies on brucellosis cases. The present study found higher MPV, PDW and RDW in brucella arthritis patients than control group.

Increased CRP and ESR have been reported to be involved in active inflammation and are often considered as useful criteria for the diagnosis and follow-up effectiveness of treatment in brucella and other inflammatory conditions [1, 17, 18]. The studies evaluating the correlation between MPV and CRP, ESR and SAT reported different results. Kader et al. [19] found a significant negative correlation between MPV and SAT. Öztürk et al. [20] found a negative correlation between MPV and CRP, while two studies found positive correlation between MPV and CRP [21, 22]. The present study found a significant positive correlation between NLR and MPV, whereas there was no significant correlation between MPV, SAT, ESR and CRP.

Our study has several limitations. Firstly, it is a retrospective study with a relatively small sample size and synovial fluid was not taken from all patients. It would be very useful if we can compare the markers between patients with brucella arthritis, septic arthritis and reactive arthritis. The studies with a larger number of patients and more comprehensive analyses can provide further data on these variables.

## Conclusion

MPV, PDW, RDW, NLR and PLR values can be useful complementary indirect markers for diagnosis of BA in children. We believe that these variables can be taken into account as quick, cheap and easily measurable new inflammatory markers in patients with BA. Further prospective studies are required to externally cross-validate our findings in larger cohorts of BA patients.

## Abbreviations

BA: Brucella arthritis
CRP: C-Reactive protein
ESR: erythrocyte sedimentation rate
Hb: hemoglobin
MPV: mean platelet volume
NLR: neutrophil to lymphocyte ratio
PDW: platelet distribution width
PLR: platelet to lymphocyte ratio
PLT: platelet
RDW: red cell distribution width
SAT: standard agglutination test
WBC: white blood cell
